# Primary Cooking Fuel Choice and Respiratory Health Outcomes among Women in Charge of Household Cooking in Ouagadougou, Burkina Faso: Cross-Sectional Study

**DOI:** 10.3390/ijerph16061040

**Published:** 2019-03-22

**Authors:** Adama Sana, Nicolas Meda, Gisèle Badoum, Benoit Kafando, Catherine Bouland

**Affiliations:** 1 Département de santé publique, Université Ouaga 1 Pr Joseph Ki-Zerbo, Avenue Charles de Gaulle, Zogona, Ouagadougou 03 BP 7021, Burkina Faso; nicolas.meda@gmail.com (N.M.); benikaf@yahoo.fr (B.K.); 2 Centre de Recherche en Santé Environnementale et Santé au Travail, Ecole de Santé Publique, Université Libre de Bruxelles, route de Lennik 808, 1070 Brussels, Belgium; catherine.bouland@ulb.ac.be; 3 Unité de Formation et de Recherche en Sciences de la Santé (UFR/SDS), Université Ouaga 1 Pr Joseph Ki-Zerbo, Avenue Charles de Gaulle, Zogona, Ouagadougou 03 BP 7021, Burkina Faso; gisebad@yahoo.fr; 4 Service de pneumo-phtisiologie, Centre Hospitalier Universitaire Yalgado Ouédraogo, Ouagadougou 03 BP 7022, Burkina Faso

**Keywords:** cooking fuel, biomass, respiratory health, women, Burkina Faso

## Abstract

*Background*: Approximately 3 billion people, worldwide, rely primarily on biomass for cooking. This study aimed to investigate the association between respiratory symptoms among women in charge of household cooking and the type of fuel used for cooking. *Methods*: A community-based cross-sectional survey was conducted. A total of 1705 women that were randomly selected, completed the survey. We also performed a bivariate and a multivariate analysis to verify the possible associations between respiratory symptoms in women in charge of household cooking and the type of cooking fuel used. *Results*: Dry cough, breathing difficulties, and throat irritation frequencies were statistically high in biomass fuel users when compared to liquefied petroleum gas (LPG) users. It was also the case for some chronic respiratory symptoms, such as sputum production, shortness of breath, wheezing, wheezing with dyspnea, wheezing without a cold, waking up with shortness of breath, waking up with coughing attacks, and waking up with breathing difficulty. After adjustment for the respondents’ and households’ characteristics; dry cough, breathing difficulties, sneezing, nose tingling, throat irritation, chronic sputum production, wheezing, wheezing with dyspnea, wheezing without a cold, waking up with shortness of breath, waking up with coughing attacks, and waking up with breathing difficulty were symptoms that remained associated to biomass fuel compared to LPG. Women who used charcoal reported the highest proportion of all the chronic respiratory symptoms compared to the firewood users. However, this difference was not statistically significant except for the wheezing, waking up with coughing attacks, and waking up with breath difficulty, after adjustment. *Conclusion*: Exposure to biomass smoke is responsible for respiratory health problems in women. Charcoal, which is often considered as a clean fuel compared to other biomass fuels and often recommended as an alternative to firewood, also presents health risks, including increased respiratory morbidity in women. Effective and efficient energy policies are needed to accelerate the transition to clean and sustainable energies.

## 1. Introduction

The International Energy Agency (IEA) estimates that there are approximately 3 billion people worldwide that still rely on the traditional use of solid biomass, such as firewood, charcoal, agricultural waste, or animal dung, for cooking meals [1]. In 2030, 2.3 billion people will still lack access to clean cooking facilities [1]. For economic, but also cultural and social reasons [2], biomass-based fuels remain at the top of the list of the preferred energy for cooking in several developing countries. In sub-Saharan Africa, more than 90% of households rely on wood, charcoal, and waste for cooking.

The biomass fuels are often burned in poorly ventilated kitchens and/or in traditional cookstoves characterized by very low efficiency and a high emission of pollutants. Indeed, the smoke emitted during biomass combustion contains toxic components that include a high concentration of particulate matter (PM), carbon monoxide (CO), nitrogen oxides (NOX), sulphur oxides (SOX), formaldehyde, and polycyclic organic matter [3,4]. In homes where biofuels are used, the rate of particulate matter, less than 10 micrometers diameter (PM_10_) over 24 h, is usually between 300 and 3000 micrograms per meter cube (µg/m^3^) and can sometimes rise up to 10,000 µg/m^3^ during the cooking activity [4]. These rates largely exceed the recommended health threshold values. In 2000, household air pollution (HAP) related to the use of solid fuels was classified amongst the ten major risk factors for global health, and the fifth risk cause of Disability Adjusted Life Years (DALYs) in low-income countries [5].

Many studies have highlighted the link between indoor air pollution and the occurrence of various health problems, including cardiovascular and respiratory diseases in the short and medium term, as well as chronic exposures [6,7,8,9,10,11]. Air pollution emitted from the burning of solid fuels is estimated to be responsible for 4.6% of the global burden of disease (GBD) [12]. According to the World Health Organization, household air pollution from biomass burning is responsible for about 4.3 million of global premature deaths worldwide [13].

Often in charge of family food cooking, women are exposed to sometimes high concentrations of harmful pollutants contained in the smoke [3,4,11]. According to the GBD 2017, a significant decrease in the health risks related to household air pollution was reported [14]. Notwithstanding, household air pollution has moved from the 9th leading risk in 2007 to the 12th leading risk to women a decade later, probably related to economic development and local initiatives, with a geographical variation [14].

In Burkina Faso, respiratory illness is the second reason why outpatients consult physicians and the fourth reason why women aged ≥15 years are hospitalized, after malaria, anemia, and abortion complications [15].

Although biomass-based fuels remain the main cooking fuel used in many West African countries, there are few studies on their association to respiratory diseases/symptoms for women living in this part of the world, especially in the urban areas. Moreover, most of these studies do not distinguish the health effects of smoke emanating from different types of biomass fuels, in particular, the difference between the effects of wood and charcoal. Indeed, charcoal, firewood, or agricultural crop residues are all biomass fuels. However, studies have shown differences in the concentration of emitted pollutants. For example, Taylor E.T. and Nakai S., in their study conducted in 2011 in rural and peri urban Sierra Leone, found that the suspended particulate matter mean concentration in the kitchens of homes using wood stoves was significantly higher than in the kitchens using charcoal stoves: Mean (SD) = 882.4 (518.0) versus 197.2 (136.0) µg/m^3^, with a *p*-value equal to 0.003 [16]. Ingale L.T. et al., in rural India, showed that PM_10_ concentration was higher in households using agro waste when compared to firewood: 4423 ± 2793 vs. 6285 ± 3996 µg/m^3^ [17]. 

This study aimed to investigate the relationship between biomass use as a primary cooking fuel and the respiratory symptoms experienced by women in charge of household cooking. Particularly, we aimed to investigate whether there was a statistically significant difference in respiratory symptoms prevalence amongst women who burnt biomass fuels compared to women who used clean fuels, such as liquefied petroleum gas (LPG), and furthermore, if there was a difference in the health effects between wood and charcoal used as the main cooking fuel.

## 2. Materials and Methods

### 2.1. Study Area

Burkina Faso is a landlocked country with a Sudano-Sahelian climate characterized by two seasons: a dry season that lasts for about 8 months and a rainy season of 4 months (May–June to September), during which rainfall averages between 600 mm and 800 mm. Firewood and charcoal are the main energy sources used by households. The central region, where Ouagadougou is located, is the main center for the consumption of timber forest products and non-timber forest products (NTFPs) in the country. In urban Ouagadougou, 43.93% of the households use wood as the primary fuel for cooking, whilst 15.60% use charcoal and 40.41% use gas [18]. Thus, 59.53% of households rely on biomass-based fuels as the primary cooking fuel.

The concentration of pollutants in kitchens is important in Ouagadougou, particularly CO concentration, which at times exceeds the WHO 1- and 8-h recommended values, 2010, of 35 mg/m^3^ (25 ppm) for 1 h and 10 mg/m^3^ (10 ppm) for 8 h [19]. In 2002, the number of deaths attributed to domestic pollution was estimated at 21,500 in Burkina Faso [20].

The study took place at Ouagadougou, during the dry season, in three urban neighborhoods: Kilwin, Tanghin, and Tampouy. The choice of these sites is explained by their diversified socio-spatial characteristics, which are representative enough of all the commune of Ouagadougou.

### 2.2. Study Design and Participants

A community-based cross-sectional survey was conducted from 7 March to 4 April 2017. This study was part of a larger research project (IRDC project #107347) focused on the relationship between indoor air pollution and respiratory diseases in Ouagadougou. Eligible participants were women or girls aged at least 18 years, with a minimum of 2 years of residence in the neighborhood.

No existing sampling frame was available. Therefore, for sampling, the interviewers moved to a central point in each of the selected neighborhoods. Then, three directions corresponding to the number of pairs of interviewers, were randomly selected. Interviewers included all households to the right which met the inclusion criteria of their respective direction. Once at the end of the street, each investigator turned right and included all the households on the left, and vice versa until the desired sample size was obtained. In each concession (extended family house or multifamily home), only one household was selected. Then, the woman who was mainly in charge of cooking in the household was interviewed.

In case of refusal, the reasons were documented. Another selection had to be made in the same concession. If there were several households that were cohabiting, or another house of the same type, it would be selected and would replace the previous one. In case of the absence of the woman of the household, the interviewers went back as many times as necessary to obtain the agreement or the refusal to participate in the survey.

### 2.3. Questionnaires and Data Collection

Study participants completed a questionnaire that included:

Independent variables: the socio-demographic characteristics and socio-economic status of the household, active or passive smoking status, kitchen characteristics, type of fuels used in the home for cooking and lighting, environmental exposure (i.e., second-hand smoking, incense burning, mosquito coils burning.

Dependent variables were the health outcomes: the perceived respiratory health, history of respiratory symptoms (i.e., dry cough, breathing difficulties, throat irritation observed in the past 2 weeks, during the cooking activities, shortness of breath, effort cough, wheezing during the past year, history of chronic cough, chronic phlegm, effort chest tightness, effort dyspnea, wheezing without a cold, woken by shortness of breath, woken by coughing attacks, or woken by breathing difficulty, or having an asthma crisis during the past year and asthma being diagnosed by a health professional). A validated questionnaire was used to obtain information about the respiratory symptoms [21,22,23,24]. The symptoms were broadly grouped into two groups: Acute respiratory symptoms that occurred during the cooking activities over the last two weeks, and chronic respiratory symptoms notified during the past year.

The responses from face to face interviews with trained interviewers, were collected in the language of choice of the respondents, translated, and recorded in French by the interviewers. A signed informed consent was requested from all the participants before administration of the questionnaire.

The questionnaire was assessed by public health professionals from the School of Public Health of the «Université Libre de Bruxelles» and «Université Ouaga 1 Professeur Joseph KI-ZERBO». A pre-test was conducted before the start of the survey. The survey was conducted by trained interviewers.

The study was approved by the Ethics Committee for Research in Health of Burkina Faso, in deliberation N° 2015-9-114.

### 2.4. Data Analysis

Data collected were encoded using Epi-Data. Description of the sample and the analysis were performed using Stata software version 13.0 (StataCorp. 2013., College Station, TX, USA).

The analysis only considered the main fuel used by the household woman.

We compared three groups based on the answers to the survey, composed of:

- LPG as the principal cooking fuel,
- Charcoal as the principal cooking fuel,
- Firewood as the principal cooking fuel.

Furthermore, a group called “biomass”, which included both firewood users and charcoal users was compared to the LPG group.

We used conventional statistics to describe the sample. Independent variables were described using relative frequencies (percentages) and the median (with standard deviation). Pearson chi-square was used to verify the possible association of the reported respiratory symptoms with the type of fuel used. We performed bivariate and multivariate logistic regressions to analyze the associations between the primary cooking fuel and the respiratory outcomes, with an adjustment for potential confounding variables, such as age, level of education, cooking duration, kitchen location, household size, exposure to secondhand smoke at home and at work, socioeconomic status, exposure to mosquito coils and incense burning smoke, road traffic exposure, and cooking stove improvement. A separate model was used for each outcome.

A *p* value < 0.05 was considered significant.

Detailed information on the energy preference for cooking in the study group, study population sampling, and the sociodemographic characteristics has previously been reported in Reference [18].

## 3. Results

A total of 1734 households representing the same number of women in charge of cooking in their household were sampled for this study. Finally, 1705 participants who had completed the questionnaire were considered for this analysis.

### 3.1. Sociodemographic Characteristics

The sample for the survey (Table 1) varied from 18 to 85 years of age, with a median of 36 years, where 59.53% used biomass as the primary fuel for cooking and about 44% only used firewood. About 85% of the households studied used at least a combination of two types of fuel for cooking (households cooking fuel preferences and the factors associated with cooking fuel choice were developed in another paper). There were no smokers; however, 31.68% of women lived with at least one smoker and 11.81% were exposed to tobacco smoke in their workplace. Table 1 summarizes the characteristics of the survey sample according to the main type of cooking fuel.

### 3.2. Health Outcomes

The reported respiratory outcomes from the different cooking fuel user groups allowed the drawing up of the following description (Table 2). Women who used biomass reported a higher proportion of all the chronic respiratory symptoms compared to LPG users (Table 2). Amongst the biomass users, charcoal users reported a higher proportion of all the chronic respiratory symptoms compared to firewood users (Table 2). However, in the bivariate analysis, this difference was statistically significant for waking up with coughing attacks, *p* = 0.012 (Table 2).

In the bivariate analysis, dry cough, breathing difficulties, and throat irritation frequencies were statistically higher for biomass fuel users compared to LPG users, with *p*-values = 0.001, 0.002, and 0.000, respectively. It was also the case for some chronic respiratory symptoms, such as chronic phlegm, shortness of breath, wheezing, wheezing with dyspnea, wheezing without a cold, waking up with shortness of breath, waking up with coughing attacks, and waking up with breathing difficulty (Table 2). Meanwhile, self-reported asthma, *p* = 0.142, and chronic cough proportion, *p* = 0.096, were not significantly different between the two groups (LPG versus biomass), as was the case for effort dyspnea, asthma crisis, and coughing during effort (Table 2). Respiratory health perception was different between the two groups with a better perception amongst the LPG users (Table 2). This perception did not significantly differ between the charcoal and firewood users. 

A multivariate analysis was carried out to investigate the relationship between the cooking fuel and the different symptoms that were studied. An adjustment was made for age, level of education, cooking duration, kitchen location, household size, exposure to secondhand smoke at home and at work, socioeconomic status, exposure to mosquito coils and incense burning smoke, road traffic exposure, and cooking stove improvement. After adjustment, the results presented in Table 3 show that dry cough, breathing difficulties, throat irritation, chronic sputum production, wheezing without a cold, woken by shortness of breath, woken by coughing attacks, and woken with breathing difficulty remained associated with biomass fuels compared to LPG use.

Charcoal use was associated with wheezing, waking by coughing attacks, waking with breathing difficulty, when compared to firewood users (Table 3). Watery eyes during cooking were most frequent among firewood users, *p* = 0.012.

In addition, cooking for more than 2 h was significantly associated with breathing difficulties during cooking, with an adjusted odds ratio (aOR): 1.56 (1.16–2.09); nose tingling aOR: 1.57 (1.19–2.05); throat irritation aOR: 1.46 (1.16–1.84); effort cough aOR: 1.35 (1.08–1.70); effort chest tightness aOR: 1.31 (1.06–1.61); wheezing with dyspnea aOR: 1.43 (1.06–1.92); and waking with breathing difficulties aOR:1.50 (1.15–1.95) (Table S1).

Respiratory outcomes were mostly reported amongst women who reported biomass use with an improved cooking stove compared to the LPG stove users’ group (Table S1). Acute respiratory outcomes were most frequent amongst firewood with improved cooking stove users compared to the LPG users (Table S1). However, chronic respiratory symptoms were mostly reported by charcoal with improved cooking stove users when compared to the LPG users (Table S1).

## 4. Discussion

The study aimed to profile respiratory symptoms that were linked to domestic cooking fuel in Ouagadougou city using a cross-sectional study design.

Cross-sectional surveys are studies at the level of evidence 4, and are not aimed at establishing a causal relationship. The strength of the results is strongly dependent on the method for selecting the participants. The possible biases at this level are selection bias (non-representativeness of the sample in relation to the target population), ranking bias which may be due to a poor definition of the exposure variables (i.e., exposure measure through indoor air pollutants concentration was not carried out, a combination of two or three types of fuels by several women), to the respondent (memorization bias), and confusion bias. The methodology used in our study integrated these biases to reduce their impact as much as possible, i.e., via the size of the sample, the selection of the participants, the adjustment of potential factors of confusion. Although the potential confounding variables, such as age, education, socioeconomic status, etc., were controlled for, other parameters were not considered, which may have also impacted the variable of interest (e.g., Body mass index, other relevant exposure).

Data were collected during the dry season. The season could have had an impact on our results. For example, Buchner and Rehfuess noticed that acute lower respiratory infection (ALRI) frequency in children was higher during the dry season than during the rainy season, and that the effects of cooking fuel types on child ALRI were lower during the dry season [25]. Burkina Faso is a sub-Saharan African country, characterized by a dry tropical climate, that alternates between a short rainy season and a long dry season, and it includes parts of the Sahel in the north of the country. In Burkina Faso, during the dry season, dust storms occur frequently. The dust-laden trade wind called the Harmattan causes dry skin, cracked lips, and can irritate the airways. Sahara dust storms can carry particulate material, pollutants, and potential allergens (e.g., heavy metals, pesticides, spores, fungi, bacteria, etc.) [26]. The Saharan dust particles have significant implications on human respiratory health, even more so because the particulate loadings can far exceed healthy levels [26].

However, data for all the participants were collected during the dry season, as the symptoms prevalence could be increased in the same way for all the participant groups. The comparison between groups should not have been influenced by that.

Despite these limitations, the study still provided additional information to support a change in fuel choice.

The major strengths of this study were its size and the adjustment for numerous confounders.

This study found a high prevalence of reported acute and chronic respiratory symptoms amongst women using biomass fuel as the main cooking fuel compared to LPG users in urban Ouagadougou.

Kurmi et al., in their study conducted on Nepalese populations, also reported that women exposed to biomass were more likely to complain of wheezing (32.0% versus 23.5%) and breathlessness (17.8% versus 12.0%) (vs. LPG), where they reported higher frequencies compared to our study [27]. In the same way, Siddiqui et al. found more throat pain, coughing, coughing with sputum, and breathing difficulties among wood users in comparison to LPG users, with even higher proportions than those observed in our study [28].

However, Desalu and collaborators noticed a lower prevalence of coughing, wheezing, and breathlessness in both the biomass and non-biomass groups [29].

These differences could be explained by an under-reporting of some respiratory symptoms (wheezing, breathlessness, and bringing up phlegm) which were often considered as normal by people [27], and the difference in the background levels of pollution.

Regarding chronic cough and chronic phlegm, Kurmi and collaborators found similar proportions in both the biomass and non-biomass exposed groups, respectively, and a lower prevalence in the biomass-using group [27].

Our analysis highlighted that the use of biomass fuel was associated with some acute and chronic respiratory symptoms. These results were consistent with other studies [27,29,30].

Biomass fuel use and asthma diagnosis have been studied by Regalado et al. [31]. Similar to our study, Regalado et al. found no significant difference between biomass and non-biomass users [31]. At the same time, even though the difference was not significant, asthma diagnosis and asthma crisis were less frequent among biomass users. As the present study only considered the main current fuel used by the participant, this result could be explained by a probable change in the cooking fuel because of illness, through self-awareness, or on the advice of a health professional or any third person.

Other findings of the study were that respiratory outcomes were mostly reported amongst biomass with improved cookstove users compared to the LPG stove users group. Even if the initial goal of the improved cookstove was to minimize the fuel consumption and smoke emissions, many of the improved stoves that are available on the local market are not able to protect the users from pollutants resulting from the burning of biomass [32,33,34].

The study also reported that wheezing, waking up from coughing attacks, and waking up with breathing difficulty were significantly negatively associated with charcoal use (versus firewood). Such a comparison between wood and charcoal has not been analyzed to that extent.

Charcoal is often considered as a clean fuel compared to the other biomass fuels, such as firewood, animal dung, crop residues, etc. [35,36]. Nevertheless, we found significant protective effects on the prevalence of wheezing and being woken by coughing attacks in firewood users compared to charcoal users. Even if there was no significant difference between firewood and charcoal concerning chronic cough and shortness of breath, our findings suggested that charcoal use significantly increased the risk of more chronic respiratory symptoms (4/5 on our list and 2/5 after adjustment) compared to firewood (2/5 and 0/5 after adjustment). Das et al. also found positive associations between respiratory symptoms and firewood compared to charcoal [37]. Charcoal, even if relatively smoke-free, may still present risks to human health [38]. Moreover, charcoal is not easy to set on fire. For that reason, the cook usually needs to use a fire starter often made with waste materials, such as plastic bags, tire rubber cut or inner tire tubes, drainage oil, paper, agricultural waste, petrol coke powder, twigs, or dry herbs. Most of these materials are not free from harmful chemicals and pollutants when burned. In our sample, more than 64% of the respondents reported using plastic bags to light the fire and 8.5% used rubber or the inner tire tube. However, it was highlighted that plastic (commonly derived from petrochemicals) burning releases toxic gases like dioxins, furans, mercury, and polychlorinated biphenyls, as well as some additives as phthalates and brominated flame retardants which pose health risks, especially to human lungs [39]. Nevertheless, charcoal due to its low smoke emission appears to be harmless, and users tend to stay close to the cookstove during the cooking activity. However, it produces large amounts of carbon monoxide (CO), which that can have harmful effects even at low exposure, by increasing the carboxy-hemoglobin (COHb) concentration.

## 5. Conclusions

Exposure to biomass smoke is responsible for the respiratory health problems of women that are in charge of cooking. Charcoal, which is often considered as a clean fuel compared to other biomass fuels and is often recommended as an alternative to firewood, also presents health risks including an increased respiratory morbidity in women.

Effective and efficient energy policies are needed to accelerate the transition to clean and sustainable energies.

## Figures and Tables

**Table 1:** Socio-demographic characteristics of the participants, *n* = 1705.

Variables	Respondents by Type of Main Cooking Fuel				
	LPG	Charcoal	Firewood	Biomass *	Overall
Age in year, mean (SD)	33.4 (11.0)	34.9 (11.2)	38.0 (13.5)	37.2 (13.0)	35.6 (12.4)
Age of primary cook in year, mean (SD)	11.2 (2.5)	11.2 (2.7)	10.9 (2.6)	11 (2.6)	11.1 (2.6)
Cooking time (minute/day), mean (SD)	161 (115)	164 (138)	176 (145)	173 (143)	168 (133)
Indoor kitchen, *n* (%)	142 (28.6)	10 (3.8)	11 (1.5)	21 (2.1)	395 (23.2)
Household size, mean (SD)	7 (4)	7 (5)	9 (5)	8 (5)	8 (4)
Average family monthly income (SD)	109,284 (81,684)	76,670 (63,639)	72,667 (57,709)	73,761 (59,365)	88,112 (71,381)
Smokers in the family, *n* (%)	193 (28.1)	86 (32.3)	259 (34.7)	345 (34.1)	538 (31.7)
Formal education ≤ 6 years, *n* (%)	333 (48.3)	67 (25.2)	129 (17.2)	196 (19.31	1176 (69.0)

LPG: liquefied petroleum gas; SD = standard deviation; Biomass *: firewood users’ group + charcoal users’ group.

**Table 2:** Prevalence and values of significance (*p*-value) for respiratory health outcomes by main cooking-fuel type ^#^.

Symptoms	LPG Users		Biomass Users *		*p* Value	Charcoal Users		Firewood Users		*p* Value
	*n* = 690		*n* = 1015			*n* = 266		*n* = 749		
	*n*	%	*n*	*%*		*n*	%	*n*	%	
**Acute respiratory symptoms**										
Dry cough	107	15.51	269	26.5	***<0.001***	60	22.56	209	27.9	*0.09*
Breath difficulties	75	10.87	166	16.35	***0.002***	38	14.29	128	17.09	*NS*
Throat irritation	145	21.01	295	29.06	***<0.001***	87	32.71	208	27.77	*0.128*
**Chronic respiratory symptoms**										
Chronic cough	95	13.77	170	16.75	*0.096*	52	19.55	118	15.75	*0.155*
Chronic phlegm	145	21.3	292	28.77	***0.001***	86	32.33	206	27.5	*0.136*
Shortness of breath	114	16.52	209	20.59	***0.036***	59	22.18	150	20.03	*NS*
Effort cough	166	24.06	287	28.28	*0.053*	73	27.44	214	28.57	*NS*
Wheezing	90	13.04	184	18.13	***0.005***	58	21.8	126	16.82	*0.071*
Effort chest tightness	235	34.06	387	38.13	*0.087*	107	40.23	280	37.38	*NS*
Effort dyspnea	321	46.52	493	48.57	*NS*	128	48.12	365	48.73	*NS*
Wheeze with dyspnea	76	11.01	146	14.38	***0.043***	43	16.17	103	13.75	*NS*
Wheeze without cold	49	7.1	112	11.03	***0.007***	34	12.78	78	10.41	*NS*
Woken by shortness of breath	146	21.16	288	28.37	***0.001***	83	31.2	205	27.37	*NS*
Woken by coughing attacks	105	15.22	212	20.89	***0.003***	70	26.32	142	18.96	***0.012***
Woken with breath difficulty	104	15.07	193	19.01	***0.036***	60	22.56	133	17.76	*0.087*
Asthma reported	28	4.06	28	2.76	*0.142*	10	3.76	18	2.4	*NS*
Asthma crisis	24	3.48	27	2.66	*NS*	11	4.14	16	2.14	*0.087*
**Perceived respiratory health**										
Excellent	54	8	45	4.52	*Reference*	8	3.1	37	5.01	*Reference*
Very good	103	15.26	169	16.97	***0.004***	40	15.5	129	17.48	*NS*
Good	441	65.33	659	66.16	***0.006***	176	68.22	483	65.45	*0.192*
Fair	40	5.93	55	5.52	*0.084*	13	5.04	42	5.69	*NS*
Poor	37	5.48	68	6.83	***0.006***	21	8.14	47	6.37	*0.123*

^#^ 1705 women took part in the study; LPG: liquefied petroleum gas; Biomass *: firewood users’ group + charcoal users’ group; NS = not significant; Bold number = statistically significant value.

**Table 3:** Adjusted odds ratios (by age, cooking duration, kitchen location, education level, household size, exposure to secondhand smoke at home and at work, socioeconomic status, exposure to mosquito and incense burning smoke, road traffic exposure, stove type) for respiratory health outcomes by main domestic cooking fuel.

Respiratory Symptoms	Multivariate Analysis			
	LPG ^†^ vs. Biomass * (*n* = 1636)		Charcoal ^†^ vs. Firewood (*n* = 975)	
	Adjusted OR (95% CI)	*p* Value	Adjusted OR (95% CI)	*p* Value
**Acute respiratory symptoms ^$^**				
Dry cough	**1.91 (1.45–2.54)**	**<0.001**	1.42 (1.00–2.02)	0.051
Breath difficulties	**1.57 (1.13–2.19)**	**0.007**	1.19 (0.78–1.82)	-
Throat irritation	**1.60 (1.23–2.74)**	**<0.001**	0.76 (0.54–1.05)	0.095
**Chronic respiratory symptoms ^£^**				
Chronic cough	1.17 (0.86–1.59)	0.313	0.72 (0.49–1.06)	0.097
Chronic phlegm	**1.61 (1.24–2.08)**	**<0.001**	0.80 (0.58–1.11)	0.187
Shortness of breath	1.11 (0.83–1.48)	0.496	0.80 (0.55–1.16)	-
Effort cough	1.15 (0.90–1.48)	0.265	0.97 (0.70–1.35)	-
Wheeze	**1.37 (1.01–1.87)**	**0.045**	**0.63 (0.44–0.92)**	**0.018**
Effort chest tightness	1.25 (0.99–1.58)	0.057	0.89 (0.65–1.21)	-
Effort dyspnea	1.03 (0.82–1.29)	-	1.06 (0.78–1.45)	-
Wheeze with dyspnea	1.34 (0.95–1.87)	0.091	0.71 (0.47–1.08)	-
Wheeze without cold	**1.57 (1.06–2.32)**	**0.025**	0.76 (0.48–1.20)	-
Woken by shortness of breath	**1.30 (1.01–1.69)**	**0.046**	0.78 (0.56–1.08)	-
Woken by coughing attacks	**1.46 (1.09–1.96)**	**0.010**	**0.62 (0.43–0.88)**	**0.007**
Woken with breath difficulty	**1.37 (1.02–1.85)**	**0.036**	**0.66 (0.45–0.96)**	**0.028**
Asthma reported	0.74 (0.39–1.39)	0.344	0.63 (0.24–1.60)	-
Asthma crisis	0.79 (0.42–1.51)	-	0.41 (0.17–1.00)	**0.049**
**Other symptoms ^$^**				
Burning eye	**1.93 (1.52–2.45)**	**<0.001**	1.25 (0.91–1.72)	0.162
Watery eye	**1.93 (1.54–2.41)**	**<0.001**	**1.49 (1.09–2.03)**	**0.012**

LPG: liquefied petroleum gas; Biomass *: firewood users’ group + charcoal users’ group; ^†^: reference; ^$^: Acute respiratory symptoms reported during cooking; ^£^: Chronic respiratory symptoms during the past year; Bold number = significant value.

## Supplementary Materials

The following are available online at https://www.mdpi.com/1660-4601/16/6/1040/s1, Table S1: Adjusted odds ratios for respiratory health outcomes by cooking duration and the combination fuel–stove used.
