# Supplementary material for: Primary Cooking Fuel Choice and Respiratory Health Outcomes among Women in Charge of Household Cooking in Ouagadougou, Burkina Faso: Cross-Sectional Study
Source: Int J Environ Res Public Health. 2019 Mar 22;16(6):1040. doi: 10.3390/ijerph16061040 (PMC6466344; doi:10.3390/ijerph16061040)
Supplement: Supplementary file 1 [file ijerph-16-01040-s001.pdf]

Supplementary material

Table S1. Adjusted odds ratios for respiratory health outcomes by cooking duration and the combination fuel–stove used.

**Respiratory symptoms**	**Cooking Duration ≤2hours vs >2hours°**	**Combination fuel + stove***			
		**LPG vs (charcoal + improved cookstove)**	**LPG vs (charcoal + traditional cookstove)**	**LPG vs (firewood + improved cookstove)**	**LPG vs (firewood + traditional cookstove)**
**Acute respiratory symptoms**					
Dry cough	1.15(0.90–1.47)	**1.53(1.04–2.26)**	1.56(0.61–4.02)	**2.17(1.60–2.94)**	**1.75(1.06–2.89)**
Breath difficulties	**1.56(1.16–2.09)**	1.34(0.84–2.14)	2.11(0.76–5.87)	**1.67(1.17–2.39)**	1.58(0.88–2.81)
Throat irritation	**1.46(1.16–1.84)**	**1.80(1.27–2.55)**	**3.05(1.37–6.79)**	**1.54(1.15–2.04)**	1.15(0.70–1.89)
**Chronic respiratory symptoms**					
Chronic cough	1.08(0.82–1.43)	1.44(0.95–2.17)	1.75(0.68–4.54)	1.12(0.80–1.58)	0.74(0.39–1.41)
Chronic phlegm	1.00(0.80–1.26)	**1.88(1.33–2.66)**	1.53(0.65–3.63)	**1.55(1.17–2.07)**	1.27(0.77–2.08)
Shortness of breath	0.99(0.76–1.29)	1.23(0.83–1.83)	2.21(0.94–5.22)	1.13(0.82–1.55)	0.60(0.32–1.12)
Effort cough	**1.35(1.08–1.70)**	**1.09(0.76–1.56)**	2.11(0.95–4.71)	1.12(0.84–1.48)	1.35(0.86–2.13)
Wheeze	1.08(0.83–1.42)	**1.84(1.24–2.73)**	1.78(0.69–4.62)	1.18(0.84–1.66)	1.35(0.77–2.35)
Effort chest tightness	**1.31(1.06–1.61)**	1.33(0.97–1.84)	1.82(0.83–3.99)	1.23(0.95–1.59)	1.14(0.74–1.76)
Effort dyspnea	0.94(0.77–1.16)	0.99(0.72–1.36)	1.12(0.51–2.46)	1.09(0.85–1.40)	0.94(0.61–1.44)
Wheeze with dyspnea	**1.43(1.06–1.92)**	**1.65(1.06–2.55)**	1.85(0.67–5.12)	1.22(0.85–1.77)	1.23(0.66–2.30)
Wheeze without cold	1.35(0.96–1.90)	**1.82(1.10–3.01)**	2.79(0.99–7.85)	1.51(0.99–2.32)	1.12(0.52–2.42)
Woken by shortness of breath	1.07(0.85–1.35)	**1.53(1.08–2.17)**	1.87(0.82–4.24)	1.22(0.92–1.63)	1.11(0.69–1.80)
Woken by coughing attacks	1.28(0.99–1.66)	**1.97(1.35–2.87)**	2.21(0.92–5.27)	1.19(0.86–1.65)	1.58(0.95–2.64)
Woken with breath difficulty	**1.50(1.15–1.95)**	**1.66(1.12–2.46)**	**2.56(1.07–6.11)**	1.17(0.84–1.63)	1.55(0.92–2.62)
Asthma reported	1.29(0.72–2.31)	0.92(0.38–2.21)	1.17(0.15–9.24)	0.68(0.32–1.44)	0.53(0.12–2.37)
Asthma crisis	1.02(0.56–1.85)	1.02(0.42–2.47)	**4.51(1.21–16.89)**	0.62(0.29–1.34)	0.53(0.12–2.39)
**Other symptoms**					
Burning eye	1.87(0.71–1.07)	**1.56(1.13–2.15)**	**2.51(1.14–5.53)**	**2.11(1.64–2.72)**	1.52(0.99–2.35)
Watery eye	0.92(0.75–1.13)	**1.39(1.02–1.90)**	**3.02(1.29–7.09)**	**3.24(1.74–2.87)**	**1.76(1.15–2.70)**

LPG: liquefied petroleum gas; *Adjustment for age, cooking duration, kitchen location, education level, household size, exposure to secondhand smoke at home and at work, socioeconomic status, exposure to mosquito and incense burning smoke, road traffic exposure; ° Adjustment by primary fuel type, age, kitchen location, education level, household size, exposure to secondhand smoke at home and at work, socioeconomic status, exposure to mosquito and incense burning smoke, road traffic exposure, type of stove.
